# Molecular epidemiological investigation of *Cryptococcus* spp. carried by captive koalas (*Phascolarctos cinereus*) in Japan

**DOI:** 10.1128/spectrum.02903-23

**Published:** 2024-02-27

**Authors:** Miki Omura, Kazuo Satoh, Takashi Tamura, Aya Komori, Koichi Makimura

**Affiliations:** 1 Laboratory of Medical Mycology, Graduate School of Medicine, Teikyo University, Tokyo, Japan; 2 Teikyo University Institute of Medical Mycology, Tokyo, Japan; Mycology Laboratory, Wadsworth center, Albany, New York, USA

**Keywords:** *Cryptococcus neoformans*, *Cryptococcus gattii*, multi-locus sequence typing, MLST, molecular epidemiological investigation, koala, One Health

## Abstract

*Cryptococcus neoformans* and *Cryptococcus gattii* cause cryptococcosis, a systemic mycosis that infects a wide range of species. Recent molecular biological investigations have allowed for the genotyping of these species, providing more detailed information on their pathogenicity and infection routes. Koalas (*Phascolarctos cinereus*) are frequently colonized by *Cryptococcus* spp., but molecular epidemiological studies have yet to be conducted in Japan. Here, we conducted multi-locus sequence typing (MLST) analysis on *Cryptococcus* spp. colonization isolates obtained from all koalas kept in seven parks across Japan. Out of 46 koalas examined, 10 (22%) were positive for *C. gattii* and 3 (6.5%) were positive for *C. neoformans*. All *C. gattii* isolates belonged to molecular type VGI and were either sequence type (ST) 51 or a novel ST, and all *C. neoformans* isolates belonged to molecular type VNI and ST23. Despite the frequent movement of koalas between parks, the STs were relatively park-specific, suggesting that the floor of the rearing barns is a source of infection and may act as a reservoir. MLST analysis confirmed that *C. gattii* was transported, established, and spread by koalas in areas where *C. gattii* was not originally present. MLST analysis is considered useful in assessing the pathogenicity and tracing the transmission routes of *Cryptococcus* spp. carried by koalas.

**IMPORTANCE:**

This is the first study to conduct a multi-locus sequence typing analysis on *Cryptococcus* spp. carried by captive koalas in Japan. Cryptococcosis remains a globally high-fatality fungal infection in humans, and captive koalas are known to carry a high percentage of *Cryptococcus* spp. Through this research, the molecular types and transmission routes of *Cryptococcus* spp. carried by koalas have been elucidated, revealing the potential role of enclosure flooring as a reservoir. It has been confirmed that *Cryptococcus gattii*, which is not endemic in Japan, has become established through koalas and is spreading to new individuals in Japan. This study is believed to provide valuable insights into koala conservation and contribute to the One Health approach for Cryptococcosis, a zoonotic infection.

## INTRODUCTION

Cryptococcosis is a fungal infection that occurs in many animals and humans (1). Cryptococcal meningitis affects nearly 250,000 people worldwide annually, resulting in 181,000 deaths and a 100% mortality rate if the infection goes untreated (2). *Cryptococcus* is designated as number one among the “Critical Priority Group” in the “WHO fungal priority pathogens list to guide research, development, and public health action” (3). Cryptococcosis is an opportunistic infection mainly affecting the lungs and central nervous system. *Cryptococcus* spp. are isolated from soil and decaying wood worldwide (4), and infection occurs via inhalation of the fungus (5). The koala (*Phascolarctos cinereus*) is designated as an endangered species on the Australian government’s domestic Red List, published following the Environment Protection and Biodiversity Conservation Act in 2022. Koalas have a high rate of *Cryptococcus* spp. colonization in their nasal cavities, but most are asymptomatic (6–10). Therefore, koalas in Australia are considered sentinels (i.e., the first warning sign) of an outbreak of *Cryptococcus* spp. (10). In the cryptococcosis outbreak on Vancouver Island, infection was confirmed in birds, ferrets, cats, dogs, dolphins, and other animals before humans, contributing to the early detection of the human outbreak (11, 12).

*Cryptococcus* spp. are a diverse genus of fungi in the phylum Basidiomycota, which includes a variety of species, including non-pathogenic species (4). *C. neoformans* and *C. gattii* are the primary pathogens responsible for cryptococcosis in humans and animals (4). Previously, *Cryptococcus* spp. were considered a monotypic species until *C. gattii* was reclassified in 1970 (13). With advances in molecular techniques, it became evident that this genus contains multiple species and has undergone taxonomic revisions over time (14, 15). Although the classification of *Cryptococcus* spp. remains controversial, it has been suggested that the terms *C. neoformans sensu lato* and *C. gattii sensu lato* are used (16). To divide species, various methods have been attempted, including DNA fingerprinting (17, 18), PCR fingerprinting based on microsatellite- (M13) or minisatellite-specific primers (19–22), random amplification of polymorphic DNA analysis (23–26), amplified fragment length polymorphism analysis (27, 28), restriction fragment length polymorphism analysis of the URA5 (22, 29) and PLB1 (30) genes, and the use of intergenic spacer (IGS) sequences (31). These methods supported the classification of *C. neoformans* into molecular types VNI–IV and VNB, while *C. gattii* is divided into molecular types VGI–IV. Furthermore, multi-locus sequence typing (MLST) analysis assigns these species into ~600 sequence types (STs) each (32). Genotyping with these molecular tools allows for the evaluation of cryptococcal pathogenicity and monitoring of the local and global distribution of these species.

*C. neoformans* is distributed in soil worldwide, and VNI is the most prevalent molecular type (33). By contrast, *C. gattii* inhabits decaying trees, especially Australian eucalypts (34), and it was initially distributed in Oceania and tropical to subtropical regions (35). However, in recent years, the distribution of *C. gattii* has expanded to temperate areas (35). In the 2000s, an outbreak of *C. gattii* emerged on Vancouver Island, Canada, and disseminated to North America (36, 37). The pathogens in this outbreak were identified as highly pathogenic strains of *C. gattii* (VGIIa, VGIIc) (37, 38). In Japan, *C. neoformans* causes most clinical cases of human cryptococcosis, and cryptococcosis caused by *C. gattii* has been observed exclusively among patients with a history of overseas travel (39–41). However, the first case of cryptococcosis caused by *C. gattii* VGIIa was identified in a patient with no travel history to Canada or the USA in 2007 (42), and this raised concern about the possibility of highly pathogenic *Cryptococcus* spreading in Japan.

Over the past two decades, we have investigated cryptococcosis among koalas in Japan in a series of studies, revealing the status of carriage and its influence on humans and the environment (43–46). We reported the first case of *C. gattii* detected in humans and animals residing in Japan (43). However, molecular epidemiological studies using MLST have not been reported to date. Therefore, in this study, we conducted MLST analysis of *Cryptococcus* spp. carried by koalas in Japan to investigate their virulence and transmission routes from a public health standpoint.

## MATERIALS AND METHODS

### Sample collection

**Fig 1:**
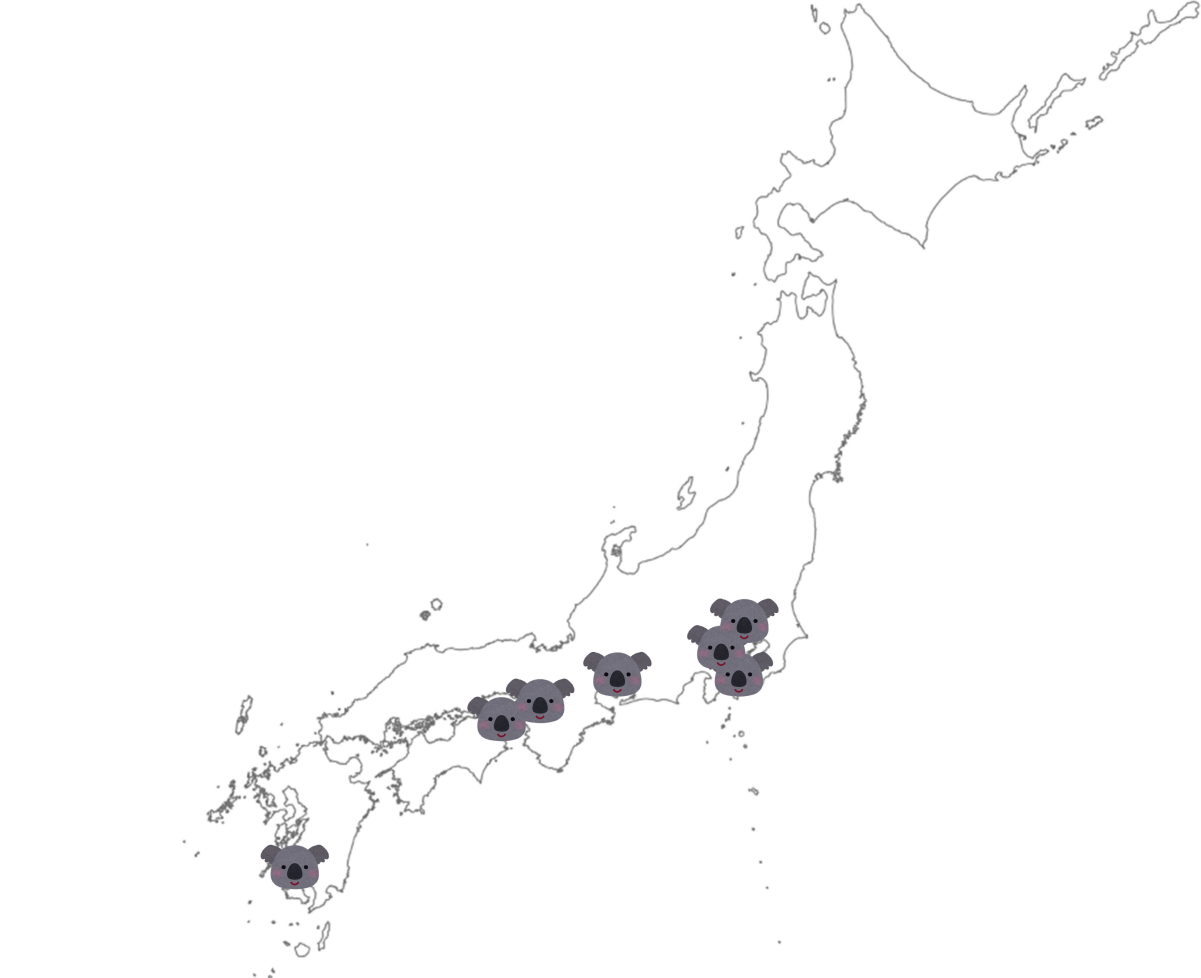
Location of the parks surveyed in this study. A koala symbol indicates the location of each park. All seven parks that keep koalas in Japan participated in the study. The locations of Parks A–G in Japan are shown. Map source: Geospatial Information Authority of Japan website (https://www.gsi.go.jp/ENGLISH/index.html). Based on “Chiriin Chizu vector” (Geospatial Information Authority of Japan; https://maps.gsi.go.jp/vector/#4.396/36.020903/135.696955/&ls = vblank&disp = 1&d = l) created by the School of Medicine, Teikyo University, Miki Omura.

Samples were obtained from koalas and their environment from August to December 2021 at seven parks in Japan (Parks A–G) (Fig. 1). Most koalas were born in Japan, but some were imported from Australian parks (Parks H–K). All 46 koalas kept in Japan were included in the survey, except for female koalas with pouch young. Koala samples were collected by swabbing the nasal cavity with a sterile swab. In addition, 47 samples were collected from locations where koalas reside and from surrounding areas with a potential presence of *Cryptococcus* spp. Floor samples were similarly collected by direct swabbing, and sand and soil samples were collected directly from the environment. The nasal cavity samples collected from six zookeepers and veterinarians were submitted simultaneously. Sampling was conducted in cooperation with the Japanese Association of Zoos and Aquariums (JAZA).

### Koala information

Information about the age, sex, family lineage, place of birth, history of transfer between parks, and cause of death for all 392 koalas kept in Japan from the start of koala rearing in 1984 until 2021 was obtained from the Japanese Studbook for Koala provided by JAZA.

### Culture and identification

Sterile swab samples were promptly inoculated into a micafungin-coated CHROMagar Candida plate (Kanto Chemical, Tokyo, Japan) (46) and sent to our institute. Sand and soil samples were sent directly to our institute, then 1 g of the sample was suspended in 5 mL of saline solution and allowed to stand for 30 min. Then, 200 µL of the supernatant was inoculated onto the plates described above, which were incubated at 37°C. The resulting yeast colonies showing white mucoid-like growth were selected and identified by matrix-assisted laser desorption/ionization time-of-flight mass spectrometry (MALDI-TOF MS: BioTyper, Bruker Daltonics, Bremen, Germany). The database version of MALDI-TOF MS was DBUpdate_V4.0.0.1_4613–5627.

### MLST analysis

DNA was extracted from the isolates identified as *C. neoformans* or *C. gattii* by MALDI-TOF MS using the method of Makimura et al*.* (47). MLST was performed according to the International Society for Human and Animal Mycology (ISHAM) consensus scheme (32). The PCR cycle was modified for samples that could not be sequenced as follows: an initial denaturation step at 95°C for 3 min, then 12 cycles of 95°C for 30 s, 62°C–56°C (with a decrease of 2°C every two cycles) for 30 s, and 72°C for 1 min, followed by 25 cycles of 95°C for 30 s, 56°C for 30 s, and 72°C for 1 min. Seven loci (*CAP59*, *GPD1*, *LAC1*, *SOD1*, *PLB1*, *URA5*, and *IGS1*) were amplified by PCR and sequenced. The obtained sequences were matched to the ISHAM-MLST database (https://mlst.mycologylab.org) to determine the allele type (AT). Then, the molecular type and sequence type were determined from the AT combinations in the same database.

### Phylogenetic analysis

A concatenated sequence of the seven loci was used for phylogenetic analysis. Data on STs commonly reported in multiple countries were obtained from the ISHAM-MLST database for global epidemiological analysis. MEGA11 software (48) was used for the analysis, with 1,000 bootstrap replicates performed, and estimations were conducted using the maximum-likelihood method based on the Tamura–Nei model (49, 50).

### Statistical analysis

Statistical analyses were conducted for sex, age, and the mother-to-child relationship of koalas. Fisher’s exact test was used for sex and the mother-to-child relationship, while the Mann–Whitney *U* test was used for age using EZR software (51).

## RESULTS

The study comprised 99 samples, including 46 from koalas, 47 from the environment, and 6 from humans. The koalas comprised 15 males and 31 females aged 0–24. *C. neoformans* was identified in 5 samples and *C. gattii* was identified in 13 samples (Table 1). Among the koalas, 3 (3/46, 6.5%) were positive for *C. neoformans* and 10 (10/46, 22%) were positive for *C. gattii* (Table 2). However, only one koala (Koala No. 2) exhibited symptoms of cryptococcosis. There was a tendency for a higher positive rate in older and male koalas, but there were no statistically significant differences regarding age or sex (age, *P* = 0.26; sex, *P* = 0.30). Moreover, there was no significant association between the cryptococcal positivity of a mother and its young (*P* = 0.197, Table 3).

**TABLE 1:** List of the samples used in this study samples^*a*^

Park	Sampling site	Koala no.	Age	Sex	Isolation	Strain no.
A	Koala	1	8	F	–	–
	Koala	2	6	M	*C. gattii*	4155
	Koala	3	4	F	–	–
	Koala	4	1	F	–	–
	Perch	–	–	–	*C. gattii*	4156
	Perch	–	–	–	–	–
	Perch	–	–	–	–	–
	Floor	–	–	–	–	–
	Floor	–	–	–	–	–
	Floor	–	–	–	–	–
	Human	–	–	–	–	–
B	Koala	5	11	F	*C. gattii*	4183
	Koala	6	11	F	*C. gattii*	4184
	Koala	7	9	M	*C. gattii*	4180
	Koala	8	7	F	*C. gattii*	4186
	Koala	9	4	M	–	–
	Koala	10	3	F	*–*	–
	Koala	11	3	F	*–*	–
	Koala	12	1	F	*C. gattii*	4189
	Koala	13	0	F	*C. gattii*	4187
	Koala	14	0	M	*–*	–
	Perch	–	–	–	*–*	–
	Perch	–	–	–	*–*	–
	Sand	–	–	–	*C. gattii*	4202
	Sand	–	–	–	*–*	–
	Sand	–	–	–	*–*	–
	Eucalyptus	–	–	–	–	–
	Eucalyptus	–	–	–	–	–
C	Koala	15	10	M	*C. gattii*	4158
	Koala	16	5	F	–	–
	Koala	17	4	F	*C. gattii*	4243
	Koala	18	2	F	–	–
	Koala	19	2	F	–	–
	Koala	20	1	M	*C. gattii*	4159
	Koala	21	1	F	–	–
	Koala	22	1	F	–	–
	Koala	23	1	M	–	–
	Koala	24	0	M	–	–
	Koala	25	0	F	–	–
	Perch	–	–	–	–	–
	Perch	–	–	–	–	–
	Perch	–	–	–	–	–
	Eucalyptus container	–	–	–	–	–
	Fan	–	–	–	–	–
	Human	–	–	–	*C. gattii*	4244
	Human	–	–	–	–	–
	Human	–	–	–	–	–
D	Koala	26	10	F	–	–
	Koala	27	7	F	–	–
	Koala	28	5	M	–	–
	Koala	29	4	F	*–*	–
	Koala	30	3	M	*C. neoformans*	4164
	Koala	31	2	F	*–*	–
	Koala	32	2	F	*–*	–
	Koala	33	2	F	–	–
	Perch	–	–	–	*–*	–
	Eucalyptus	–	–	–	–	–
	Human	–	–	–	–	–
	Human	–	–	–	–	–
E	Koala	34	6	F	*C. neoformans*	4171
	Koala	35	3	M	*C. neoformans*	4173
	Koala	36	3	F	*–*	–
	Koala	37	2	F	–	–
	Koala	38	2	F	–	–
	Perch	–	–	–	–	–
	Perch	–	–	–	–	–
	Floor	–	–	–	*C. neoformans*	4174
	Floor	–	–	–	*C. neoformans*	4176
	Floor	–	–	–	*–*	–
	Sand	–	–	–	*–*	–
	Sand	–	–	–	–	–
	Sand	–	–	–	*–*	–
	Eucalyptus	–	–	–	–	–
F	Koala	39	6	M	–	–
	Koala	40	5	M	–	–
	Koala	41	4	F	–	–
	Koala	42	2	F	–	–
	Perch	–	–	–	–	–
	Perch	–	–	–	–	–
	Perch	–	–	–	–	–
	Perch	–	–	–	–	–
	Floor	–	–	–	–	–
	Floor	–	–	–	–	–
	Floor	–	–	–	–	–
	Soil	–	–	–	–	–
	Soil	–	–	–	–	–
	Soil	–	–	–	–	–
G	Koala	43	24	F	–	–
	Koala	44	13	Ｆ	–	–
	Koala	45	12	M	–	–
	Koala	46	7	M	–	–
	Perch	–	–	–	–	–
	Perch	–	–	–	–	–
	Sand	–	–	–	–	–
	Sand	–	–	–	*–*	–
	Eucalyptus	–	–	–	–	–
	Eucalyptus	–	–	–	–	–
	Refrigerator	–	–	–	–	–
	Air cleaner	–	–	–	*–*	–

^*a*^ All koalas in Japan participated in the study, except female koalas with pouch young. There were 99 samples in total, including 46 from koalas, 47 from the environment, and 6 from humans. *C. neoformans* was detected in 5 samples, and *C. gattii* was detected in 13 samples.

**TABLE 2:** Positive rate of *Cryptococcus* spp. by sample type^*a*^^,*b*^

	*C. gattii* (+)	*C. neoformans* (+)	Total
Koala	10 (22)	3 (6.5)	46 (100)
Environment	2 (4.3)	2 (4.3)	47 (100)
Human	1 (17)	0 (0)	6 (100)
Total	13 (13)	5 (5)	99 (100)

^*a*^ *Cryptococcus* spp*.* was detected in all three sample types. *Cryptococcus* spp. was detected in about 29% of all koalas. Four environmental samples tested positive for *Cryptococcus*, including two floor samples, one sand sample, and one perch sample. *C. gattii* was detected in one human sample, but that individual did not show any *Cryptococcus*-related symptoms.

^*b*^ Data are presented as *n* (%).

**TABLE 3:** The relationship between a mother and its young in terms of the cryptococcal positivity rate in koalas^*a*^^,^^*b*^

*Cryptococcus* spp.	Child (+)	Child (−)	Total
Mother (+)	4 (31)	18 (70)	22 (48)
Mother (−) or not tested	9 (69)	15 (30)	24 (52)
Total	13 (100)	33 (100)	46 (100)

^*a*^ There was no significant difference in the cryptococcal positivity of mothers with cryptococcal-negative and -positive young, providing evidence against mother-to-child transmission.

^*b*^ Data are presented as *n* (%).

Environmental samples were extensively examined for possible cryptococcal sites and originated from 21 floors and floor coverings, 17 perches, 5 eucalyptus trees, 2 air conditioners, 1 refrigerator, and 1 eucalyptus container. Among the environmental samples, two *C. neoformans* (2/47, 4.3%) and two *C. gattii* (2/47, 4.3%) isolates were detected. Two of the isolates were detected from floor samples, one was from a floor covering sample, and one was from a perch. This detection rate of *Cryptococcus* spp. in captive koala environments was relatively lower than that reported in four Australian zoos (13%–70%) (52). These differences may be attributed to the fact that in Australian studies, eucalyptus, the ecological niche for *C. gattii* (53, 54)*,* was the subject of investigation. The eucalyptus provided to koalas in Japan is domestically produced, and in our past surveys, *Cryptococcus* spp. has not been detected in domestic eucalyptus. Furthermore, Japanese parks thoroughly clean their breeding houses, such as wiping down perches and regularly changing flooring materials, which may have contributed to the low detection rate. In addition, one human sample was positive for *C. gattii* (1/6, 17%), but there was only one colony count, and no cryptococcosis-related symptoms were observed in that individual.

**Fig 2:**
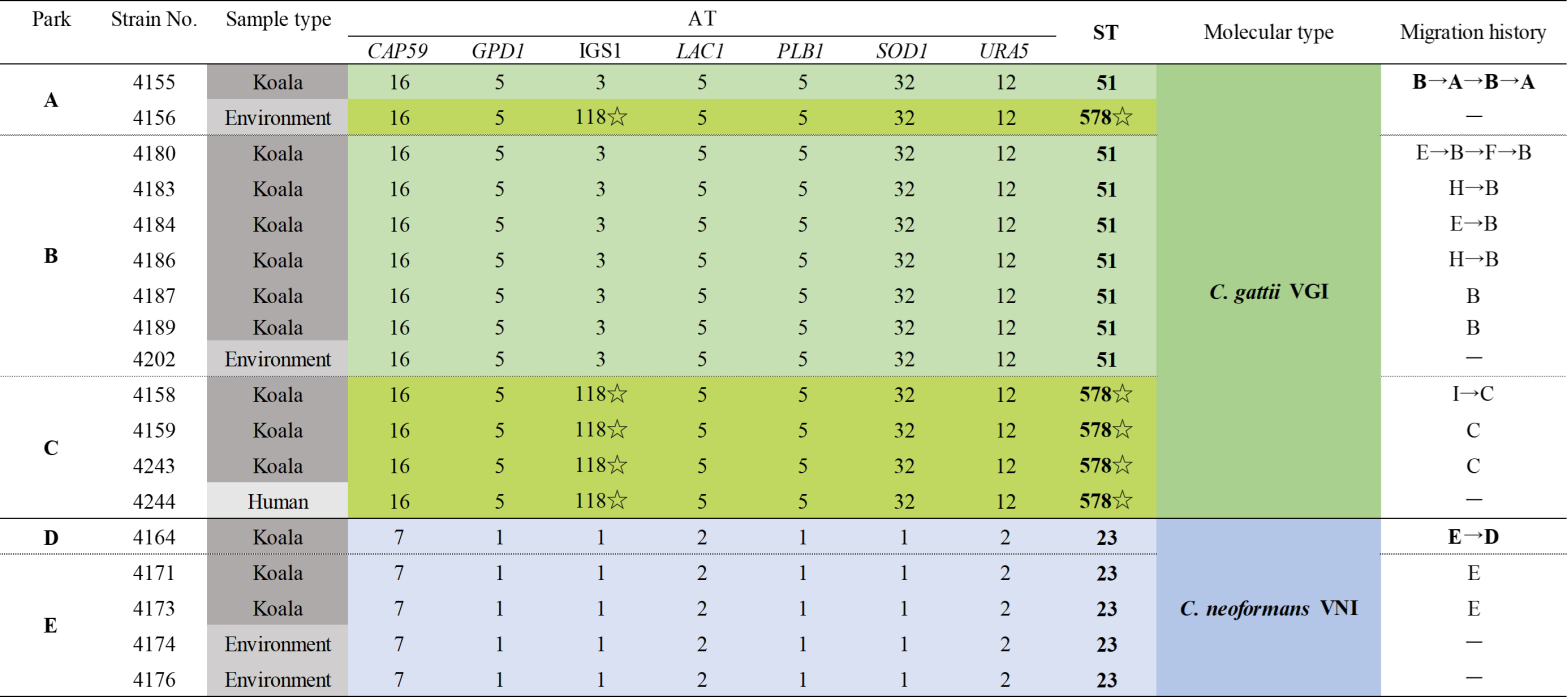
MLST data and the migration history of koalas. The molecular type of the eight *C. gattii* isolates detected from Parks A and B was VGI, ST51. The five *C. gattii* isolates detected from Parks A and C were VGI, novel ST. The molecular type of the five *C. neoformans* isolates detected from Parks D and E was VNI, ST23. The ST distribution was relatively park-specific, and the same ST tended to be detected in the same park irrespective of the collection source. ☆ indicates the newly detected AT and ST, which were assigned to AT118 and ST578. Park H and I are located in Australia.

**Fig 3:**
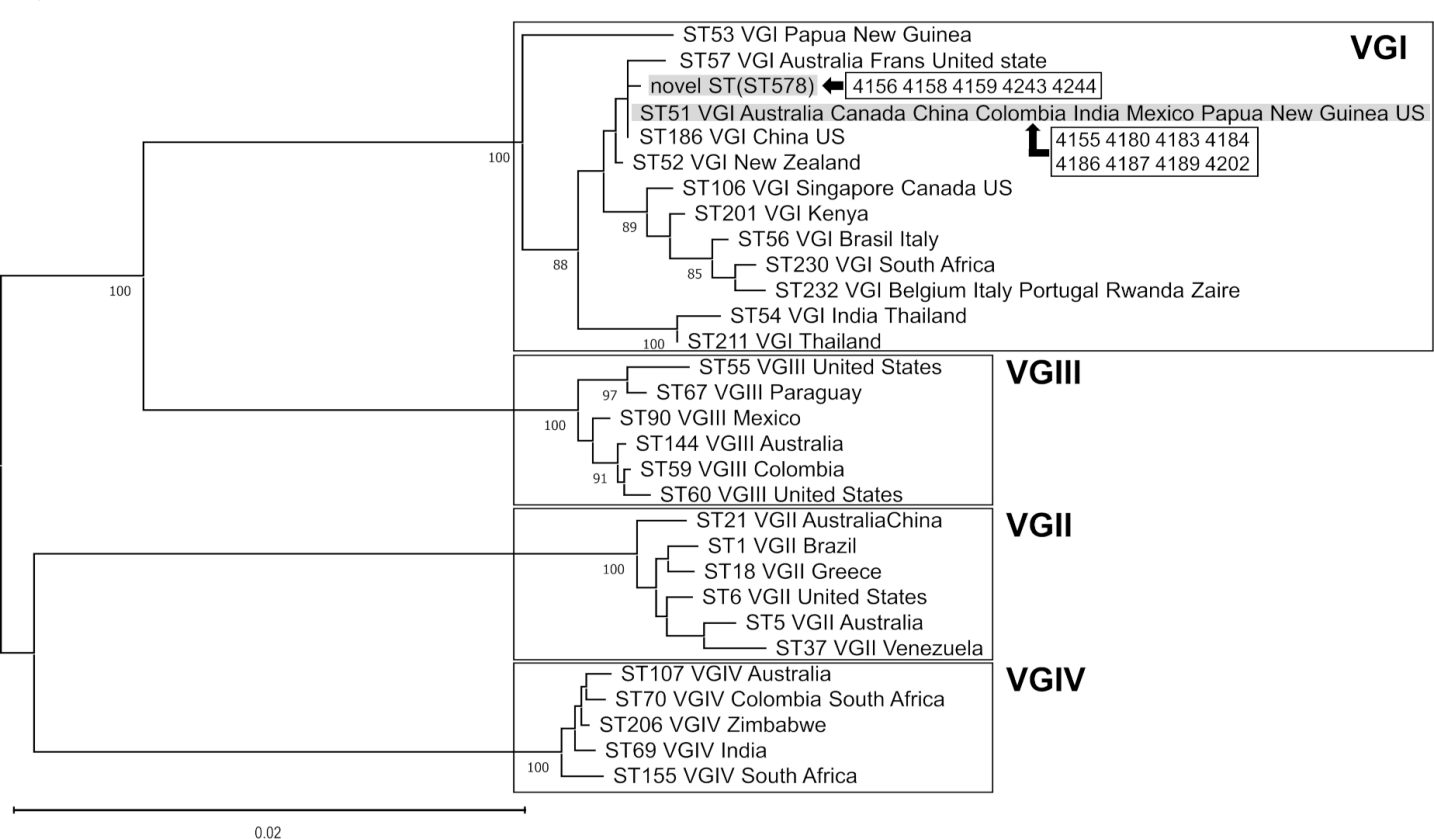
Phylogenetic tree created with the *C. gattii* isolates detected in this study and the typical ST. Information on the typical ST sequences and their countries of isolation was obtained from the ISHAM-MLST database (https://mlst.mycologylab.org). For phylogenetic analysis, the sequences of seven loci were used based on the maximum-likelihood method and the Tamura–Nei model with 1,000 bootstrap replicates. All *C. gattii* strains detected in this study belonged to VGI.

**Fig 4:**
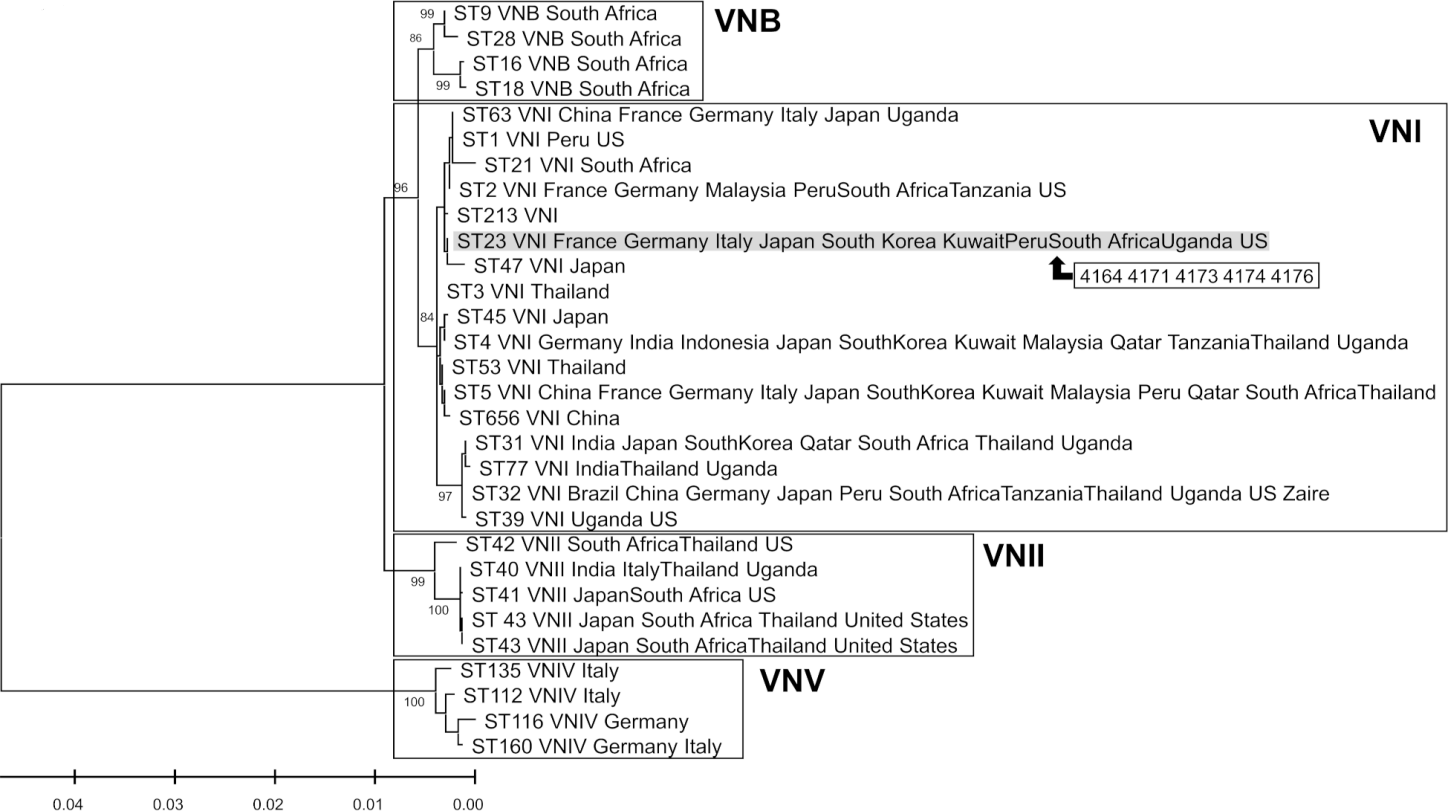
Phylogenetic tree created with the *C. neoformans* isolates detected in this study, the typical ST, and STs previously detected in Japan. Information on the typical ST and STs previously detected in Japan was obtained from the ISHAM-MLST database (https://mlst.mycologylab.org), and the analysis was performed as described in Fig. 3. All *C. neoformans* strains detected in this study belonged to VNI.

MLST was conducted on all 18 cryptococcal strains isolated from koalas, the environment, and a human (Fig. 2). All *C. gattii* isolates were of molecular type VGI, and eight isolates were of ST51 and five isolates had a novel ST. The isolates with a novel ST also possessed a novel AT that differed by two bases at the IGS1 locus of ST51. Phylogenetic analysis classified the novel ST as belonging to VGI (Fig. 3). The novel AT was submitted to the ISHAM-MLST database and assigned as 118, and the novel ST was assigned as 578. The molecular type of the five *C. neoformans* strains was VNI, ST23 (Fig. 4). The ST distribution was relatively park-specific, with a tendency for the same ST to be isolated in the same park, irrespective of the collection source. All sequences obtained in this study were deposited in the DNA Data Bank of Japan (DDBJ accession nos. LC770840–LC770917, LC771000–LC771047).

Park B, where *C. gattii* was most frequently identified, had 17 koalas that had been imported from Park H in Australia on 14 occasions between 1984 and 2016. Because the koalas in Park H have been reported to have a 100% *C*. *gattii* positive detection rate (8), it is thought that *C. gattii* was transmitted to Park B from Park H. Four of the six koalas at Park B for whom *C. gattii* was detected were born in Japan, indicating that infection occurred in Japan. Despite the close contact between a mother and its young, no association with cryptococcal positivity was detected in this study. This supported our previous finding (46) that excluded mother-to-child transmission of *Cryptococcus* spp. The same ST was detected in the environment and the koalas in Park B, suggesting that the infection might have spread through the environment rather than through koala-to-koala contact. *C. gattii* ST51 was identified in both Parks A and B. The koala in Park A (Koala No. 2), which was positive for *C. gattii*, was born in Park B and repeatedly moved between Parks A and B for mating. Therefore, it was presumed that this koala was infected in Park B. Park C imported five koalas from Parks I and J in Australia in 2013 and 2017. The novel ST may therefore have originated from either park. Park A received one koala from Park C in 2015. This koala was born in Japan, tested positive for *C. gattii*, and died of lymphoma in 2019. It is therefore possible that this koala transmitted the novel ST from Park C to Park A.

The two *C. neoformans*-positive koalas in Park E were born in Park E and remained at that park. Park E imported three koalas from Park I in Australia in 2014. *C. neoformans* may therefore have been imported from Australia at this time, but since *C. neoformans* is endemic in Japan, it could also have originated in Japan. To investigate further, additional surveys were conducted on soil and decaying wood at 16 sites other than the koala breeding house in Park E, but no *Cryptococcus* spp. were isolated (data not shown). *C. neoformans* ST23 was also detected in a koala at Park D (Koala No. 30). This koala was born in Park E and moved to Park D and may therefore have been infected in Park E.

## DISCUSSION

This study is the first nationwide survey and MLST analysis of *Cryptococcus* spp. in captive koalas in Japan. According to the Japanese Studbook for Koalas, since koala breeding began in Japan in 1984, 392 koalas have been kept in Japan, and 13 of them have died from Cryptococcosis, with the last death occurring in 2013. The mortality in cryptococcosis in koalas in Japan was 3.3% (13/392), while previously reported mortality rates were 10.7% (3/28) for captive koalas (55), 1.57% (2/127) for free-ranging koalas (56), and 0% (0/519) for wild koalas (57). This suggests that koalas have a higher mortality rate for cryptococcosis in captivity than in the wild. However, this may be because, in captive koalas, there are no causes of death, such as traffic accidents or animal attacks, which are common in wild koalas (57), and more lethal diseases like chlamydia (58) are treated in captive settings. In this study, the proportion of koalas positive for *C. gattii* or *C. neoformans* was approximately 29% (13/46), whereas in our previous studies conducted over 20 years (2 October 2001–27 May 2021), 57% (59/103) of koalas tested positive for either *Cryptococcus* at least once (to be discussed in another article). Although not directly comparable because of the different survey methods, it appears that the positive detection rate of *Cryptococcus* spp. in koalas in Japan may have decreased. In previous reports by other researchers, the positive detection rate of *Cryptococcus* spp. among captive koalas varied from 6.6% to 100% (6, 8–10, 45, 59). One study revealed that the detection rate was lower among free-ranging koalas kept under conditions that were closer to their natural environment (6.6%, 12/181) (10), suggesting that *Cryptococcus* spp. detection rates may be an indicator of breeding conditions.

*C. gattii* is not considered endemic in Japan (42), and the *C. gattii* detected in koalas is considered to have originated from Australia. In Australian studies, it was reported that *C. neoformans* was isolated more frequently from human clinical samples (60), whereas koalas were more often colonized by *C. gattii* (8–10, 35) because of their close association with eucalyptus, an environmental niche for *C. gattii* (61). The molecular type of *C. gattii* was mainly VGI in eastern Australia, with a higher population density, whereas VGII was mainly restricted to Western Australia and the Northern Territory (60, 62). All Japanese parks surveyed in this study, except for Park G, imported koalas from parks in eastern Australia, potentially explaining why only VGI was detected in this survey. *C. gattii* ST51 is one of the most widely distributed STs in the world (63–66). In two recent reports, ST51, ST57, ST154, ST159, ST188, ST366, ST395, ST396, ST459, ST460, and ST461 of VGI were isolated from 22 koalas in Australia, and ST51 was the most frequently isolated (59, 65).

*C. neoformans* is endemic in Japan, and most human clinical strains of *C. neoformans* in Japan are VNI, ST5 (67–69) [note that in some reports (68, 70), ST5 was labeled as ST46 because of the differences in the MLST database used]. Similarly, ST5 is predominantly detected in many East Asian countries, including China, Korea, and Hong Kong (69, 71–73). ST23 has not been detected in Japanese human clinical isolates but has been detected in soil in Japan (69). ST23 has also been reported to be isolated from a wide range of regions, including Europe, North and South America, Asia, and Africa (69, 74, 75). Although, to our knowledge, there have been no reports of its isolation in Australia. In Australia, *C. gattii* is the predominant *Cryptococcus* spp. carried by koalas, and the nose of the koala is thought to act like an air sampler that collects *Cryptococcus* spp. from its surroundings (10). Therefore, ST23 detected in this study may be of Japanese origin, but further research is needed to confirm this.

Parks F and G, where no *Cryptococcus* spp. was detected in this study, had unique characteristics to their breeding methods that were not found in the other parks. Park F was the only park to use concrete flooring that was cleaned with running water, whereas all the other parks used soil or sand as flooring material. *Cryptococcus* spp. is known to proliferate in soil containing bird feces (76) because the feces contains creatinine (77) that is used by the *Cryptococcus* spp. Koala urine contains higher creatinine concentrations than the urine of humans or canines (78–80), suggesting that *Cryptococcus* spp. may grow in soil or sand soaked with koala urine, which may thereby act as a reservoir. Recently, naturalistic enclosures have been recommended from an animal welfare perspective (81), and concrete floors tend to be avoided. However, the use of concrete should be reconsidered under conditions where sufficient dilution of urine cannot be expected.

Park G was the only park that bred the Victoria koala bloodline and did not exchange koalas for breeding with other parks that bred the Queensland koala and New South Wales bloodline. This restriction on koala movement may have helped prevent cryptococcosis.

There was a significant change in the ratio of prevalence of *C. gattii* to *C. neoformans* between previous surveys and this study. Over the last 20 years, *C. neoformans* has been detected around twice as frequently as *C. gattii* (33/103 koalas for *C. neoformans*, 16/103 koalas for *C. gattii*, and 10/103 koalas for both. In the cases of 10 koalas where both were detected, it was not a simultaneous infection of *C. gattii* and *C. neoformans*; each was detected in separate tests conducted at different points in time. This will be discussed in another article). By contrast, in this study, *C. gattii* was detected three times more frequently than *C. neoformans*, and symptoms of cryptococcosis were observed in only one koala infected with *C. gattii*. This may be because previous studies primarily tested symptomatic koalas. Due to the lack of health status data on koalas in previous surveys, the accurate ratios of colonization, subclinical disease, and clinical disease remain unknown. However, in the past, tests were basically conducted when koalas suspected of having cryptococcosis occurred, leading to a tendency for a higher proportion of symptomatic koalas to be tested. On the other hand, all koalas, regardless of their health status, were tested in the current study. This may indicate that *C. neoformans* is more symptomatic than *C. gattii*. In humans, *C. neoformans* typically cause infections in immunocompromised individuals, such as HIV-infected patients (82). Immunosuppression caused by koala retrovirus (KoRV) may therefore contribute to the development of *C. neoformans* infection. KoRV is associated with leukemia, lymphoma, and immunodeficiency-like diseases, and it has been reported that 60% of koalas in Japan were also infected with KoRV-B (83). Further research is needed on the relationship between cryptococcosis and KoRV.

Highly pathogenic strains, such as VGIIa (38, 84) and VGIIc (37), are not considered widespread in Japan and were not detected in this survey. However, climate change (85, 86) and increased migration of humans and animals (87, 88) may change the virulence and distribution of pathogens, and caution is therefore needed. Infectious diseases can be spread between animal species and humans, and monitoring pathogens across species is a critical element of the One Health approach.
